# Online hate network spreads malicious COVID-19 content outside the control of individual social media platforms

**DOI:** 10.1038/s41598-021-89467-y

**Published:** 2021-06-15

**Authors:** N. Velásquez, R. Leahy, N. Johnson Restrepo, Y. Lupu, R. Sear, N. Gabriel, O. K. Jha, B. Goldberg, N. F. Johnson

**Affiliations:** 1 Institute for Data, Democracy and Politics, George Washington University, Washington, DC 20052 USA; 2 ClustrX LLC, Washington, DC USA; 3 Physics Department, George Washington University, Washington, DC 20052 USA; 4 Department of Political Science, George Washington University, Washington, DC 20052 USA; 5 Department of Computer Science, George Washington University, Washington, DC 20052 USA; 6 Google LLC, Mountain View, CA USA

**Keywords:** Health care, Physics

## Abstract

We show that malicious COVID-19 content, including racism, disinformation, and misinformation, exploits the multiverse of online hate to spread quickly beyond the control of any individual social media platform. We provide a first mapping of the online hate network across six major social media platforms. We demonstrate how malicious content can travel across this network in ways that subvert platform moderation efforts. Machine learning topic analysis shows quantitatively how online hate communities are sharpening COVID-19 as a weapon, with topics evolving rapidly and content becoming increasingly coherent. Based on mathematical modeling, we provide predictions of how changes to content moderation policies can slow the spread of malicious content.

## Introduction

In addition to its health and economic effects, the COVID-19 pandemic is playing out across the world’s online platforms^1–4^. While limiting the spread of infection through social distancing, isolation has led to a surge in social media use and heightened individuals’ exposure to increasingly virulent online misinformation. Users share misinformation about prevention and treatment, making it difficult for individuals to tell science from fiction. As individuals react emotionally in their online posts to the growing death toll and economic peril^5^, online extremists are rebranding their conspiracy theories around current events to draw in new followers^6^. This growth in hateful online activity may have contributed to recent attacks against vulnerable communities and government crisis responders^7–9^.

Mitigating malicious online content will require an understanding of the entire online ecology. A rich literature across many disciplines explores the problem of online misinformation^10–14^, detailing some suggestions for how social media platforms can address the problem^1,15–19^. However, the bulk of existing work focuses on the spread of misinformation *within a single* platform, e.g., Twitter, but contemporary social media platforms are not walled gardens.

As we show in this paper, mitigating malicious online content requires an analysis of how it spreads *across multiple* social media platforms. Each social media platform is in some ways its own *universe*, i.e., a commercially independent entity subject to particular legal jurisdictions^20,21^, but these universes are connected to each other by users and their communities. We show that hate communities spread malicious COVID-19 content across social media platforms in ways that subvert the moderation attempts of individual platforms. Moreover, there is now a proliferation of other, far-less-regulated platforms thanks to open-source software enabling decentralized setups across locations^22^. Cooperation by moderators across platforms is a challenge because of competing commercial incentives; therefore we develop implications for policing approaches to reduce the diffusion of malicious online content that do not rely on future global collaboration across social media platforms^23–25^.

## Design and results

To gain a better understanding of how malicious content spreads, we begin by creating a map of the network of online hate communities across six social media platforms. We include actively moderated mainstream platforms—Facebook, VKontakte, and Instagram—that have and enforce (to varying degrees) policies against hate speech, as well as the less-moderated platforms Gab^26^, Telegram^27^, and 4Chan^28^. We focus on the distinction between actively moderated and less-moderated platforms while acknowledging that they also vary in other important ways that are outside the scope of this paper: for example, platforms also vary in terms of whether or not posted content is removed after a certain length of time and whether or not posts are identified as linked to specific user accounts. Our data include the most popular moderated social media platforms in the Americas and Europe (i.e., Facebook, VKontakte, Instagram), as well as less-moderated networks popular with hate groups (i.e., 4Chan, Gab^26–28^). These platforms allows users to create and join groups, (e.g., Facebook fan page, VKontakte group, Telegram channel 4Chan boards) that are interest-based communities—in contrast to platforms such as Twitter or Parler that have no in-built collective accounts and are instead designed for broadcasting short messages^29–31^. We refer to all these communities as “clusters.” Within such clusters, users develop and coordinate around narratives.

Our analysis includes content from 1245 hate clusters which broadcasted 29.77 million posts between June 1, 2019 and March 23, 2020. We labeled as hateful those clusters in which 2 out of the 20 most recent posts at the time of classification include hate content. We define a post as including hate content if it advocates and/or practices hatred, hostility, or violence toward members of a race, ethnicity, nation, religion, gender, gender identity, sexual orientation, immigration status, or other defined sector of society. While the anonymity of 4Chan and the Terms of Service of Facebook make it impossible for us to determine the exact number of users, we estimate that at least 12,600 distinct users posted at least once. We parsed more than 12,100 links across the selected 6 platforms, leading into 5397 different accounts. Within this linked multiverse of clusters across platforms, we found a single dominant main component connecting 341 (i.e. 27.4%) of the classified hate clusters (see Fig. 2B).

Examples of hate clusters include neo-Nazis, fascists, white supremacists, anti-semites, Islamophobes, cis-gender male supremacists, and others. They mostly cover Europe, Africa, and the Americas and communicate in dozens of languages. Most analyses of online extremist activity focus on a single platform, but extremists, like others, simultaneously use multiple platforms for complementary functions. This redundancy helps extremist networks develop resilience across the multiverse of platforms. The more moderated platforms tend to be the largest in terms of audiences and the best suited to build relations of trust (i.e., lower anonymity and greater accountability). In contrast, the less-moderated platforms have smaller number of users, yet they allow more freedom to indulge in hateful content and some offer greater anonymity and lower accountability.

An extremist group has incentives to maintain a presence on a mainstream platform (e.g., Facebook Page) where it shares incendiary news stories and provocative memes to draw in new followers. These clusters try to approach the line dividing hate speech from other political speech, but not to cross it. Then once they have built interest and gained the trust of those new followers, the most active members and page administrators direct the vetted potential recruits towards their accounts in less-moderated platforms such as Telegram and Gab, where they can connect among themselves and more openly discuss hateful and extreme ideologies.

**Figure 1:**
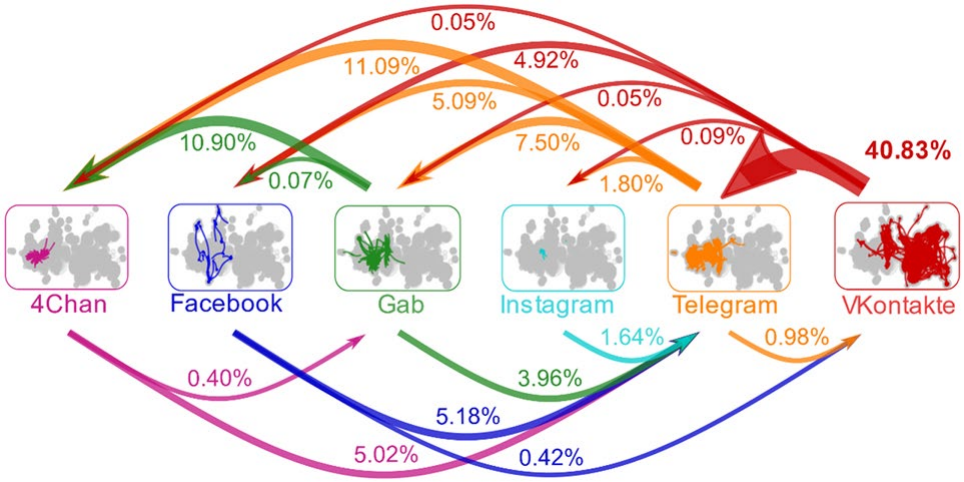
Connectivity of online hate multiverse. We counted all links between hate clusters on different social media platforms between June 1, 2019 and March 23, 2020. Each edge shows percentage of such links from hate clusters on the outbound platform to hate clusters on the inbound platform. Some platform pairs feature zero such links, hence no arrow. Although content moderation prevents users on some platforms (e.g., Facebook) from linking to some unmoderated platforms (e.g., 4Chan), users can access such content—and direct other users to it—by linking to a hate cluster on a third platform (e.g., Telegram) that then links to the unmoderated platform.

**Figure 2:**
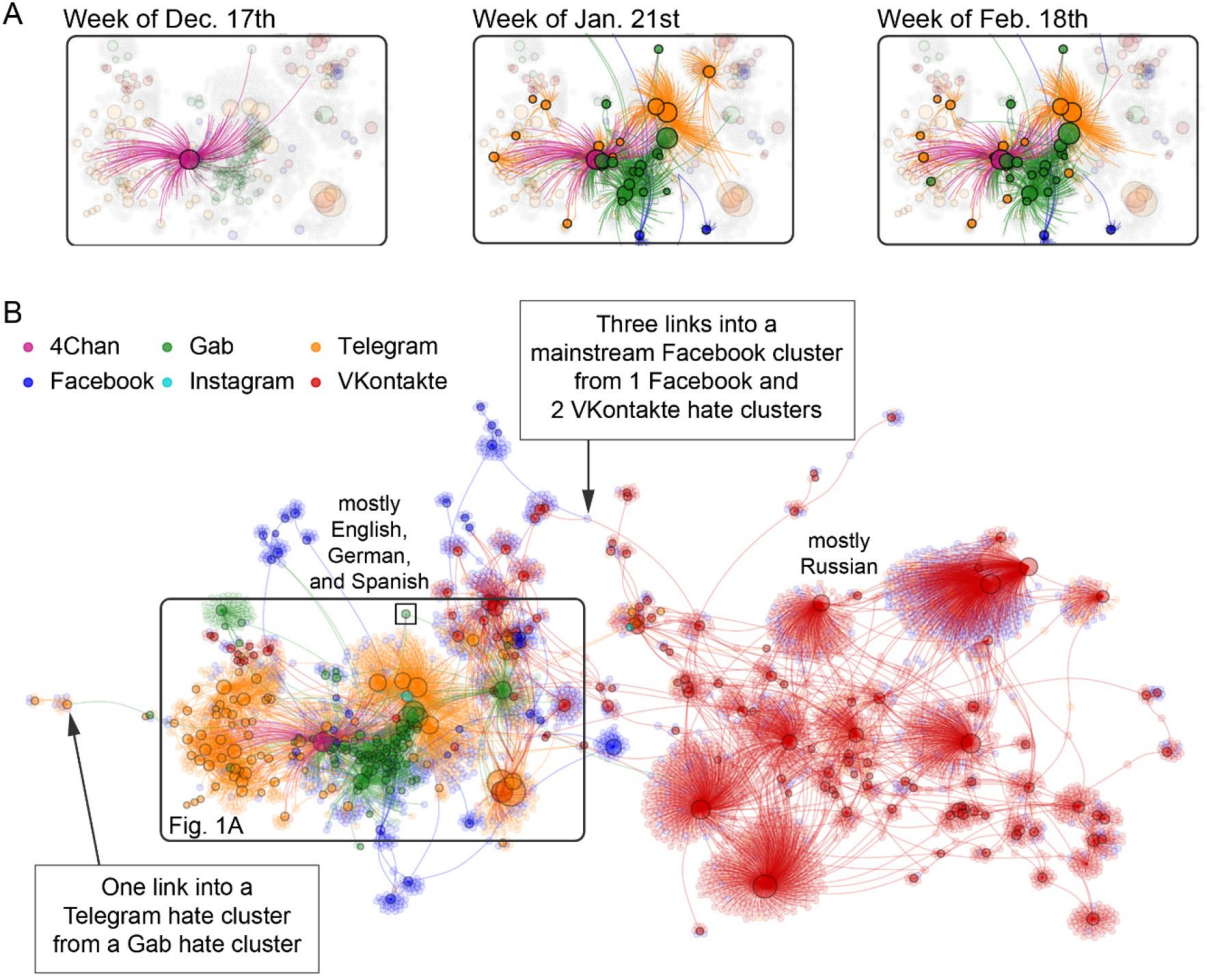
Malicious COVID-19 content spreading across the online hate multiverse. (**A**) Time evolution of birth and spread of malicious COVID-19 content within and across different social media platforms within a portion of the online hate network in (**B**) outlined in black. (**B**) The online hate multiverse comprises separate social media platforms that interconnect over time via dynamic connections created by hyperlinks from clusters on one platform into clusters on another. Links shown are from hate clusters (i.e., online communities with hateful content, shown as nodes with black rings) to all other clusters, including mainstream ones (e.g., football fan club). Link color denotes platform hosting the hate cluster from which link originates. Plot aggregates activity from June 1st, 2019 to March 23rd, 2020. The visual layout of the network emerges organically from the ForceAtlas2 algorithm such that collections of nodes appear visually closer if they are more interlinked, i.e. the layout is not pre-determined or built-in (see “Methods”). The small black square (inside the larger black square) is the Gab cluster analyzed in Fig. 3 (see “Methods” and Supplementary Information for details).

To understand more fully the dynamics by which COVID-19 content diffuses and evolves across the online hate network, and to inform the policy solutions offered, we first identified and mapped out online hyperlinks across clusters and across platforms (Figs. 1, 2) using previously published methodology^29,30^ (see description in “Methods” and Supplementary Information). Then we identified malicious content related to COVID-19 by searching for usage of specific keywords and constructs related to the pandemic. This terminology differs by time period given the quickly evolving nature of the pandemic. For example, terms such as “COVID-19” and “SARS-CoV-2” were officially introduced by the World Health Organization in February 2020. Yet, the pandemic and its effects had been discussed in these hate clusters since at least December 2019, when it was colloquially known in the clusters that we study by offensive names such as “Chinese Zombie Virus” and “Wuhan Virus”.

Figure 1 shows the percentage of links within the hate network that are between given pairs of platforms. For example, 40.83% of the cross-platform links in the network are from VKontakte into Telegram, while 10.90% of the links are from Gab into 4Chan. In part because of content moderation, only two platforms connect outward to all the other platforms: Telegram and VKontakte. For instance, Facebook automatically blocs posts with hyperlinks into 4Chan. However, a Facebook user can link to a VKontakte group or post that links into a 4Chan cluster, creating a mediated path that allow users to access misinformation and hate content across the multiverse of platforms. Figure 2 then shows how spreading occurs across these platforms: specifically, how it exploits multiple social media platforms that interconnect over time via dynamic connections created by hyperlinks from clusters on one platform into clusters on another. Each of the hate clusters appears as a node with a black circle, while other clusters linked to by hate clusters appear as nodes without black circles. The panels in Fig. 2A show how quickly malicious COVID-19 content spread between platforms and hence beyond the control of any single platform. Supplementary Tables 1 and 2 in the Supplementary Information give specific numbers for the links between all 6 platforms.

**Figure 3:**
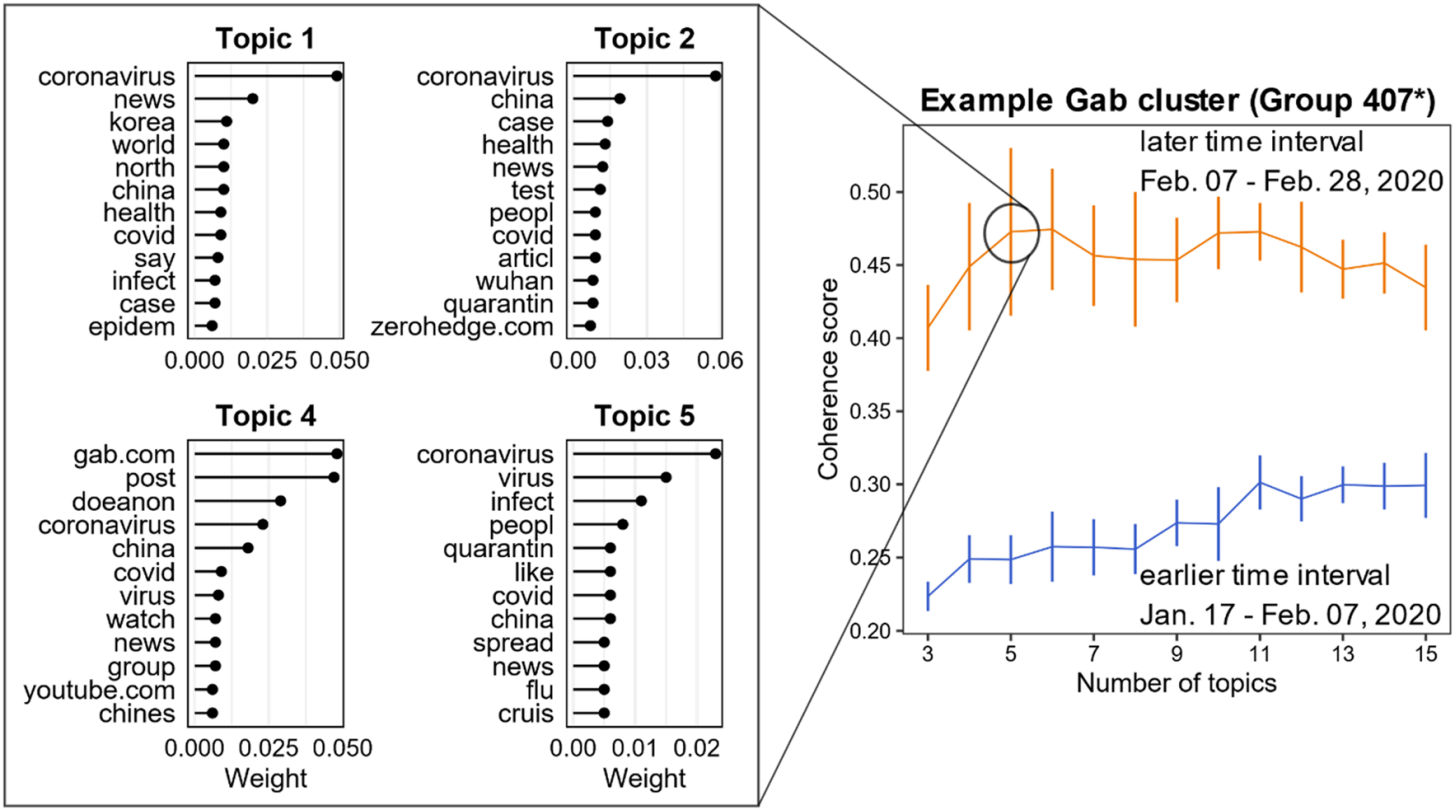
Evolution of COVID-19 content. Focusing on a single Gab hate cluster, this provides example output from our machine learning topic analysis. Although discussion of COVID-19 only arose in December 2019, it quickly evolved from featuring a large number of topics with a relatively low average coherence score, to featuring a small number of topics with high average coherence score more focused around COVID-19. As the right-hand panel shows, the discussion in this cluster became much more coherent, and focused on COVID-19, during the second 3-week period we analyzed. The right-hand panel shows the keywords in each of 5 topics discussed on this cluster during that second 3-week period. In the first three-week period, topics discussed featured profanity and hate speech such as f*** and n*****, but the conversation quickly become more focused and less like a stereotypical hate-speech rant.

We then analyze how malicious COVID-19 content evolves in the hate network following published methodology^32^. Specifically, we conduct machine-learning topic analysis using Latent Dirichlet Allocation (LDA)^33^ to analyze the emergence and evolution of topics around COVID-19. We then calculate a coherence score, which provides a quantitative method for measuring the alignment of the words within an identified topic^33^. The coherence score is a probability measure (between 0 and 1) that captures how often top words in topics co-occur with each other. We calculate this over time using a sliding window. Specifically, it is based on a one-set segmentation of the top words and uses normalized point-wise mutual information (NPMI) and the cosine similarity. The coherence score that we show is a simple arithmetic mean of all the per-topic coherences^33,34^. Figure 3 provides an example of the results of this analysis within a single hate cluster. We find that the coherence of COVID-19 discussion increased rapidly in the early phases of the pandemic, with narratives forming and cohering around COVID-19 topics and misinformation.

## Discussion

Overall, these findings show that links across social media platforms collectively form a decentralized multiverse that interconnects hate communities and hence allows malicious material to spread quickly across platforms beyond any single platform's control. Any given piece of malicious material can therefore quickly and unexpectedly reappear on a platform that had previously considered itself rid of that material. Without knowledge of this multiverse map of links within and between platforms, there is little chance of malicious material including misinformation and disinformation ever being effectively dealt with—no matter how much money and resources platforms and government entities might spend.

We now consider the implications more specifically. First, in order to understand the diffusion of COVID-19 and related malicious matter, we need to account for the decentralized, interconnected nature of this online network (Fig. 2). Links connecting clusters on different social media platforms provide gateways that can pass malicious content (and supporters) from a cluster on one platform to a cluster on another platform that may be very distant geographically, linguistically, and culturally. Figure 2 shows that consecutive use of these links allows malicious matter to find short pathways that cross the entire multiverse. Because malicious matter frequently carries quotes and imagery from different moments in a cluster’s timeline, these inter-platform links not only interconnect information from disparate points in space, but also time.

Second, malicious activity can appear isolated and largely eradicated on a given platform, when in reality it has moved to another platform. There, malicious content can thrive beyond the original platform’s control, be further honed, and later *reintroduced into the original platform* using a link in the reverse direction. Facebook content moderators reviewing only Facebook (i.e., blue) clusters in Fig. 2B might conclude that they had largely rid that platform of hate and disconnected hateful pages from one another, when in fact these same clusters remain connected via other platforms. Because the number of independent social media platforms is growing, this multiverse is very likely to remain interconnected via new links.

Third, this multiverse can facilitate individuals’ moving from mainstream clusters on a platform that invests significant resources in moderation, into less moderated platforms like 4Chan or Telegram, simply by offering them links to follow. As Fig. 1 illustrates, a user of mainstream social media communities, such as a child connecting with other online game players or a parent seeking information about COVID-19, is at most a few links away from intensely hateful content. In this way, the rise of fear and misinformation around COVID-19 has allowed promoters of malicious matter and hate to engage with mainstream audiences around a common topic of interest, and potentially push them toward hateful views.

Fourth, the topic analysis described in Fig. 3 shows that the discussion within the online hate community has coalesced around COVID-19, with topics evolving rapidly and their coherence scores increasing. Examples of weaponized content include narratives calling for supporters to purposely infect the targets of their hate with the virus, as well as predictions that the pandemic will accelerate racial conflict and societal collapse^35^. While these topics morph, the underlying structure in Fig. 2B is surprisingly robust, which suggests that our implications should also hold in the future.

In summary, so long as the capacity exists for users to link content across platforms (or even to inform users on one platform that content resides on other platforms) no single platform can address the problem of malicious COVID-19 content. Yet we also realize that coordinated moderation among all platforms (some of which are unmoderated) will always be a significant challenge. Even with bolstered collaboration between governments and platforms via forums like the Global Internet Forum to Counter Terrorism, removing extremist content across multiple social networking sites remains an solved problem^36^. Because alt-tech platforms and alternative media networks formed in backlash to content moderation efforts on mainstream platforms, they are unlikely to participate in such collaborations and, instead, may continue undermining content moderation by providing online infrastructure to share extremist content^37^.

**Figure 4:**
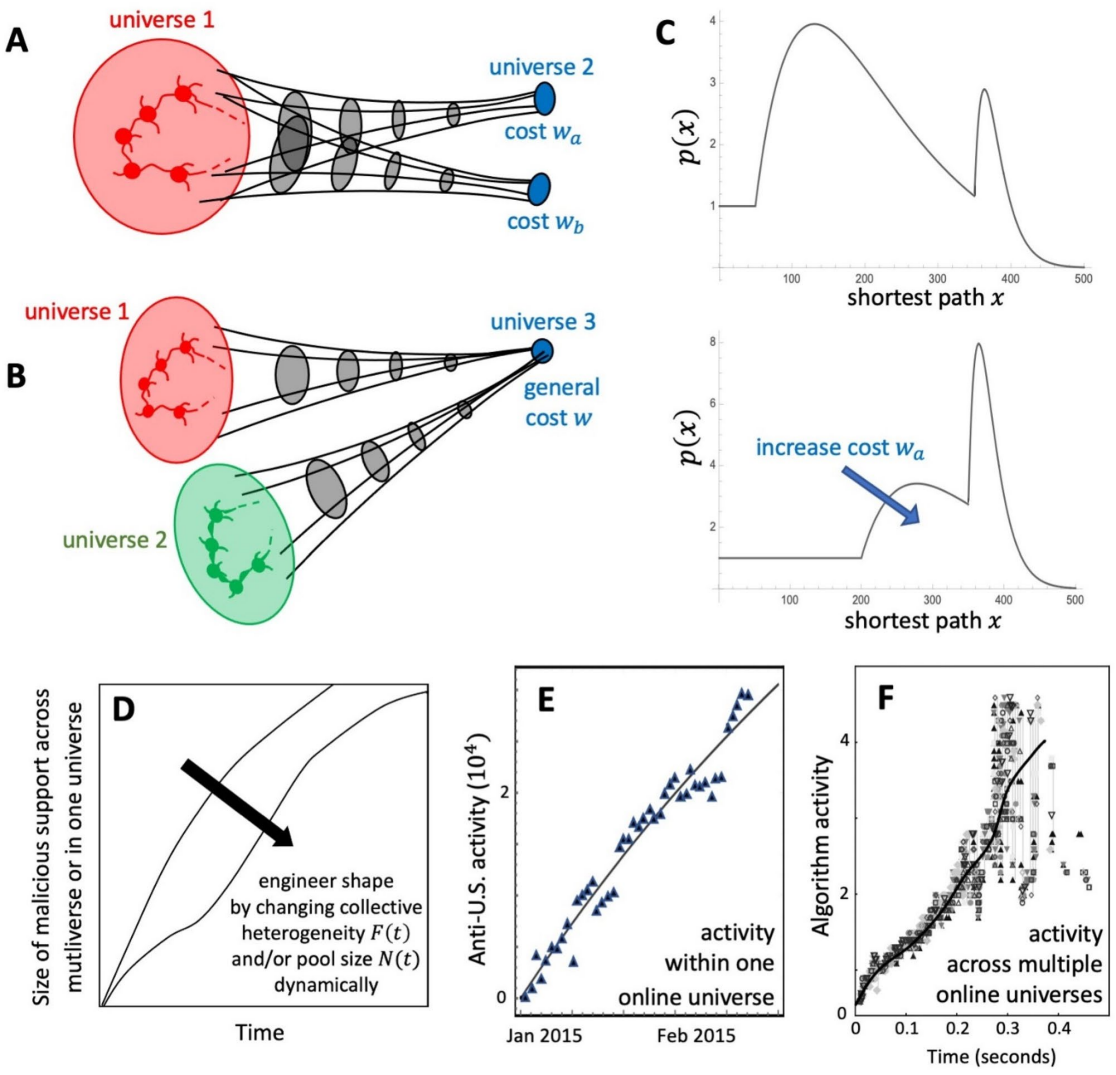
Link engineering to mitigate spreading. Details of each panel are discussed in the text with full mathematical derivations of all the predictions and results given in the Supplementary Information.

We therefore offer a mathematical model that suggests other ways for mainstream platforms to address this problem without collaborating with less-moderated platforms. Our predictions (see Supplementary Information for full mathematical details) suggest that platforms could use bilateral link engineering to artificially lengthen the pathways that malicious matter needs to take between clusters, increasing the chances of its detection by moderators and delaying the spread of time-sensitive material such as weaponized COVID-9 misinformation and violent content. This involves the following repeated process: first, pairs of platforms use the multiverse map to estimate the likely numbers of indirect ties between them. Then, without having to exchange any sensitive data, each can use our mathematical formulae to engineer the correct cost w for malicious content spreaders who are exploiting their platform as a pathway, i.e., they can focus available moderator time to achieve a particular detection rate for malicious material passing through their platform and hence create an effective cost w for these spreaders in terms of detection, shut-down, and sanctions. Figure 4A,B show typical motifs within the full multiverse in Fig. 2B. In panel C our model's mathematical prediction for motif A, shows that the distribution of shortest paths (top panel, shown un-normalized) for transporting malicious matter across a platform (i.e., universe 1) can be shifted to larger values (bottom panel) which will then delay spreading and will increase the chance that the malicious matter is detected and removed^38,39^. This is achieved by manipulating the risk that the malicious content gets detected when passing via the other platform: this risk represents a cost for the hate community in universe 1 when using the blue node(s). The same mathematics applies irrespective of whether each blue node is a single cluster or an entire platform, and applies when both blue clusters are in the same platform or are in different platforms. See Supplementary Information for case B. While Fig. 4A,B show common situations that arise in the multiverse, more complex combinations can be described using similar calculations (see Supplementary Information) in order to predict how the path lengths for hate material can be artificially extended in a similar way to Fig. 4C.

Our predictions also show that an alternative though far more challenging way of reducing the spread of malicious content, is by manipulating either (1) the size $N$ of its online potential support base (e.g., by placing a cap on the size of clusters) and/or (2) their heterogeneity $F$ (e.g., by introducing other content that effectively dilutes a cluster’s focus). Figure 4D shows examples of the resulting time-evolution of the online support, given by $N\left(1-W\left(\left[\frac{-2Ft}{N}\right]exp\left[\frac{-2Ft}{N}\right]\right)/\left[\frac{-2Ft}{N}\right]\right)$ where $W$ is the Lambert function^40^. The mathematics we develop here has implications beyond the hate network shown in Fig. 2B. Figure 4E,F show related empirical findings which are remarkably similar to Fig. 4D. Figure 4E gives an example of how an empirical outbreak of anti-U.S. hate across a single platform (VKontakte) in 2015 produces a similar shape to the upper curve in D. Finally, in panel F the pattern of an empirical outbreak for the proxy system of predatory ‘buy’ algorithms across multiple electronic platforms^41^ also produces a similar shape to lower curve in D (see Supplementary Information for details). Figure 4F is a proxy system in which ultrafast predatory algorithms began operating across electronic platforms to attack a financial market order book in subsecond time^41^. Figure 4F therefore also serves to show what might happen in the future if the hate multiverse in Fig. 2B were to become populated by such predatory algorithms whose purpose is now to quickly spread malicious matter, even if their cadence was slowed from the subsecond to the minute or hours scale in order to better mimic human behavior^11^.

Our analysis of course requires follow-up work. Our mathematical formulae are, like any model, imperfect approximations. However, we have checked that they agree with large-scale numerical simulations^38–42^ and follow similar thinking to other key models in the literature^43–45^. Going forward, other forms of malicious matter and messaging platforms need to be included. However, our initial analysis suggests similar findings for any platforms that allow communities to form. In this sense, our multiverse maps show the transport routes which need to be known and understood before what travels on this system can be controlled. We should also further analyze the temporal evolution of cluster content using the machine-learning topic modeling approach and other methods. We could also define links differently, e.g., numbers of members that clusters have in common. However, such information is not publicly available for some platforms, e.g., Facebook. Moreover, our prior study of a Facebook-like platform where such information was available showed low/high numbers of common members reflects the absence/existence of a cluster-level link, hence these quantities indeed behave similarly to each other. People can be members of multiple clusters; however, our prior analyses suggest only a small percentage are *active* members of multiple clusters. In terms of how people react to intervention, it is known that some may avoid opposing views^46^ while for others it may harden beliefs^47,48^. However, what will actually happen in practice remains an empirical question.

Finally, we do not in this work ascertain the extent to which single actors including state entities may play a more influential role in this multiverse than others, or may to some extent control it. This remains a challenge for future work, but we note that the delocalized nature of the multiverse with its multiple clusters across platforms, make it hard to understand how a single entity could control it. Our sense is, therefore, that there may well be more sinister or influential actors than others, but that there is unlikely to be any single entity in overall control. This is strengthened quantitatively by the fact that we already showed in Ref.^29^ that the size of groups in a single platform (VKontakte) has a power-law distribution which suggests some kind of organic mechanism rather than top-down control.

## Methods

Humans are not directly involved in this study. Our methodology focuses on aggregate data about online clusters and posts, hence the only data required that involves individuals is the open-source content of their public posts, which is publicly available information. Links between clusters are hyperlinks into either (a) social network account’s profiles or (b) posts hosted in these accounts’ boards. Our network analysis for Fig. 2B starts from a given hate cluster A and captures any cluster B to which hate cluster A has shared such a hyperlink.

We parsed the links and nodes signals through a combination of (a) each platform’s application programming interface and (b) parsing of the hyperlinks’ paths, parameters, and REST (representational state transfer) queries. All but one node in Fig. 2B is plotted using the ForceAtlas2 algorithm, which simulates a physical system where nodes (either hate clusters or accounts linked into by a source hate clusters but that we did not classify) repel each other while links act as springs, and nodes that are connected through a link attract each other. Hence nodes closer to each other have more highly interconnected local environments while those farther apart do not. The exception to this Force Atlas2 layout in Fig. 2B is Gab group 407* (“Chinese Coronavirus”, https://gab.com/groups/407*, see small black square in Fig. 2B) which was manually placed in a less crowded area to facilitate its visibility. This particular hate cluster was created in early 2020 with a focus on discussing the COVID-19 pandemic, but it immediately mixed hate with fake news and science, as well as conspiratorial content.

## Supplementary Information

Supplementary Information.

## Data Availability

Humans are not directly involved in this study. Aggregate information data will be provided with the Supplementary Information (SI).
