# Supplementary material for: Online hate network spreads malicious COVID-19 content outside the control of individual social media platforms
Source: Sci Rep. 2021 Jun 15;11:11549. doi: 10.1038/s41598-021-89467-y (PMC8206165; doi:10.1038/s41598-021-89467-y)
Supplement: Supplementary file 1 — Supplementary Information. [file 41598_2021_89467_MOESM1_ESM.pdf]

Supplementary Information (SI)

Paper title: Online hate network spreads malicious COVID-19 content outside the control of individual social media platforms

Authors: N. Velasquez, R. Leahy, N. Johnson Restrepo, Y. Lupu, R. Sear, N. Gabriel, O. Jha, B. Goldberg, N.F. Johnson

Contents

A. Methodology p.2

B. Theoretical description of bilateral wormhole engineering (Figs. 4A,B,C main paper) p.4

C. Theoretical description for manipulating outbreak of support surrounding malicious matter (Figs. 4D,E,F main paper) p.13

D. Example of LDA topic analysis of hate multiverse content before COVID-19 outbreak **WARNING: THIS SECTION CONTAINS OFFENSIVE MATERIAL** p.17

E. Direct and indirect links between hate clusters in different social networks p.18

A. Methodology. Our online cluster search methodology follows our earlier works referenced in the main paper (N.F. Johnson, R. Leahy, N. Johnson Restrepo, N. Velasquez, M. Zheng, P. Manrique, P. and S. Wuchty. Hidden resilience and adaptive dynamics of the global online hate ecology. Nature 573, 261 (2019); N.F. Johnson, M. Zheng, Y. Vorobyeva, A. Gabriel, H. Qi, N. Velasquez, P. Manrique, D. Johnson, E. Restrepo, C. Song, S. Wuchty, S. New online ecology of adversarial aggregates: ISIS and beyond. Science 352, 1459 (2016)), but now looking within and across multiple social media platforms. While the method can in principle be repeated for any topic, we focus in this paper on forms of hate and hate-speech defined as either (a) content that would fall under the provisions of the United States' Code regarding hate crimes or hate speech according to Department of Justice's guidelines, or (b) content that supports or promotes Fascist ideologies or regime types (i.e., extreme nationalism and/or racial identitarianism). On-line communities promoting hate have become prevalent globally and are being linked to many recent violent real-world attacks, including the 2019 Christchurch shootings. We observe many different forms of hate adopting similar cross-platform tricks. Our research avoids needing any information about individuals, just as information about a specific molecule of water is not needed to describe the bubbles (i.e. clusters of correlated molecules) that form in boiling water. We define a hate cluster for practical purposes in this paper, as a cluster (e.g., Facebook fan page, VKontakte club) in which 2 out of 20 of its most recent posts at the time of classification align with the above definition of hate. Whether a particular cluster is strictly a hate philosophy, or simply shows material with tendencies toward hate, does not alter our main findings. Links between clusters are hyper-links. Our hate cluster network analysis starts from a given hate cluster A and captures any hate cluster B to which hate cluster A has shared an explicit cluster-level link. We developed software to perform this process automatically and, upon cross-checking the findings with our manual list, were able to obtain approximately 90 percent consistency between manual and automated versions. Figure S1 shows an example of clusters and a wormhole between them, from our analysis.

Figure S1 shows the behavior within a universe (i.e., within a given platform, VKontakte) including the individual users. Gephi's ForceAtlas2 layout in Fig. S1 simulates a physical system in which nodes (clusters) repel each other while links act as springs. It is color agnostic, i.e. the color segregation emerges spontaneously and is not in-built. Nodes that appear closer to each other have local environments which are more highly interconnected while nodes that are far apart do not. Each cluster (VKontakte Group or Page) receives directly the feed of narratives and other material from that Group or Page and all members (fans) can engage in the discussions and posting activity. The visually explosive nature is similar across scales (i.e. akin to Fig. 2B of the main paper) which suggests that parts of the multiverse that are as-yet unknown or yet to be created, will exhibit similar behaviors.

The LDA (Latent Dirichlet Allocation) method is 'a generative statistical model that allows sets of observations to be explained by unobserved groups that explain why some parts of the data are similar. For example, if observations are words collected into documents, it posits that each document is a mixture of a small number of topics and that each word's presence is attributable to one of the document's topics. LDA is an example of a topic model and belongs to the machine learning toolbox and in wider sense to the artificial intelligence toolbox.' We recommend this Wikipedia entry *https://en.wikipedia.org/wiki/Latent_Dirichlet_Allocation* from which the quote comes, for useful links. We also refer to a recent paper from re-

searchers at Ghent University and Twitter, available at arXiv:1909.01436v2, titled 'Discriminative Topic Modeling with Logistic LDA', by I. Korshunova, M. Fedoryszak, H. Xiong, L. Theis, for a nice summary of new advances and analysis, as well as references therein. The coherence score is a way of measuring the alignment of the words within an identified topic. The overall coherence score is just a simple arithmetic mean of all the per-topic coherences. C_v, the coherence score, is based on a sliding window, one-set segmentation of the top words and an indirect confirmation measure that uses normalized point-wise mutual information (NPMI) and the cosine similarity. Essentially, it comprises collections of probability measures on how often top words in topics co-occur with each other in examples of the topics. The following is an example which specifically uses this C_v measure: https://ieeexplore.ieee.org/document/8259775. We refer to is for a longer-form explanation and discussion of C_v.

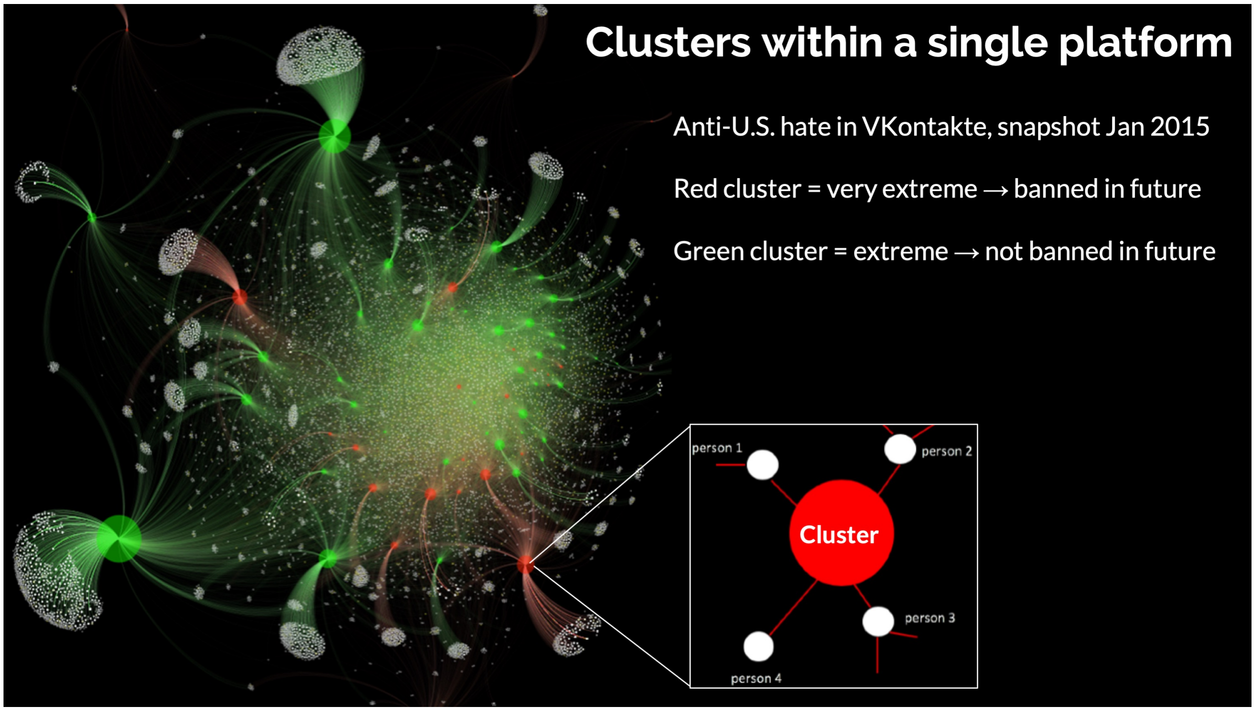

Figure S1: **Detailed snapshot shows individuals' membership in hate clusters on a single day within a single universe of anti-U.S. hate during the rise of ISIS (January 24, 2015). Red clusters are those that subsequently were shut down, while green clusters were not. White dots are individual users. The intra-universe map has similarly explosive appearance to large-scale multiverse map in Fig. 2B of the main paper. User information is not publicly available for all platforms, hence it is not included in figures in the main paper.**

B. Theoretical description of bilateral wormhole engineering (Figs. 4A,B,C main paper). This material draws heavily from the reference in the main paper: D.J. Ashton, T.C. Jarrett and N.F. Johnson, Effect of Congestion Costs on Shortest Paths Through Complex Networks, Phys. Rev. Lett. 94, 058701 (2005). We refer to that paper for demonstrations of good agreement between the core mathematics and approximations that we use, and numerical simulations – and hence numerical justifications for the approximations that we use. Each of the n nodes in a long chain (technically, a ring though a long chain also suffices) is connected to its nearest neighbors by a link of unit length. These links are directed in the 'directed' model, and undirected in the 'undirected' model. With a probability p any node can be attached to the central hub by a link of length $\frac{1}{2}$. The links to the hub are always undirected. For both the directed and undirected models, explicit expressions can be derived for the probability $P(\ell, m)$ that the shortest path between any two nodes on the ring is ℓ, given that they are separated around the ring by length m. Summing over all m for a given ℓ and dividing by $(n-1)$ yields the probability $P(\ell)$ that the shortest path between two randomly selected nodes is of length ℓ. The average value for the shortest path across the network is then $\bar{\ell} = \sum_{\ell=1}^{n-1} \ell P(\ell)$. For the undirected model, the expressions are more cumbersome because there are more paths with the same length. However, defining $nP(\ell) \equiv Q(z, \rho)$ where $\rho \equiv pn$ and $z \equiv \ell/n$, there is a simple relationship between the undirected and directed models in the limit $n \to \infty$ with $p \to 0$, i.e. $Q_{undir}(z, \rho) = 2Q_{dir}(2z, \rho)$. The models only differ in this limit by a factor of two: $z \to 2z$, with z now running from 0 to 1/2. The results which follow were obtained by generalizing this procedure. We add a cost c every time a path passes through the central hub. This cost c is expressed as an additional path-length, however it could also be expressed as a time delay or reduction in flow-rate for transport and supply-chain problems. We have in general considered three cases: (1) constant cost c where c is independent of how many connections the hub already has, i.e. c is independent of how well-used the hub is; (2) linear cost c where c grows linearly with the number of connections to the hub, and hence varies as $\rho \equiv np$; (3) nonlinear cost c where c grows with the number of pairs connected directly across the network, and hence varies as ρ^2. For a general, non-zero cost c that is independent of ℓ and m, we can write the following for a network with directed links:

$$
\begin{aligned}
P(\ell, \ell \leq c) &= \frac{1}{n-1} && (1)\\
P(\ell < m, \ell > c) &= (\ell - c)p^2(1-p)^{\ell-c-1} && (2)\\
P(\ell = m, \ell > c) &= 1 - p^2 \sum_{i-c=1}^{\ell-c-1} (i-c)(1-p)^{(i-c)-1} && (3)
\end{aligned}
$$

Performing the summation gives:

$$
P(\ell = m, \ell > c) = (1 + (\ell - c - 1)p)(1-p)^{\ell-c-1} \qquad (4)
$$

The shortest path distribution is hence:

$$
P(\ell) = \begin{cases} \frac{1}{n-1} & \forall\ \ell \leq c \\ \frac{1}{n-1}\big[1 + (\ell - c - 1)p \\ \qquad +(n-1-\ell)(\ell - c)p^2\big](1-p)^{\ell-c-1} & \forall\ \ell > c \end{cases}
$$

Using the same analysis for undirected links yields a simple relationship between the directed and undirected models. Introducing the variable $\gamma \equiv \frac{c}{n}$ with z and ρ as before, we may

define $nP(\ell) \equiv Q(z, \gamma, \rho)$ and hence find in the limit $p \to 0$, $n \to \infty$ that $Q_{undir}(z, \gamma, \rho) = 2Q_{dir}(2z, 2\gamma, \rho)$. For a fixed cost, not dependent on network size or the connectivity, this analysis is straightforward. For linear costs, dependent on network size and connectivity and for $N = 1$ central hub, we can show that there exists a *minimum* value of the average shortest path $\bar{\ell}$ as a function of the connectivity to the central hub. Hence there is an *optimal* number of connections to the central hub, in order to create the minimum possible average shortest path. We denote this minimal path length as $\bar{\ell} \equiv \bar{\ell}|_{\min}$. Such a minimum is in stark contrast to the case of zero cost per connection, where the value of $\bar{\ell}$ would just decrease monotonically towards one with an increasing number of connections to the hub. We now calculate the average shortest path, $\bar{\ell} = \sum_{\ell=1}^{n-1} \ell P(\ell)$, which yields:

$$\bar{\ell} = \frac{(1-p)^{n-c}\big[3 + (n-2-c)p\big]}{p^2(n-1)} + \frac{p\big[2 - 2c + 2n - (c-1)(c-n)p\big] - 3}{p^2(n-1)} + \frac{c(c-1)}{2(n-1)} \ . \quad (5)$$

An analytic expression for $\bar{\ell}|_{\min}$ can be obtained by setting the differential of Eq. (5) equal to zero. If n is very large, one can introduce a higher cost without compromising the minimal shortest path $\bar{\ell}|_{\min}$ since in general the nodes are already much further from one another. We can also investigate how many connections we should make for a given cost and network size, in order to achieve the minimum possible shortest path $\bar{\ell}|_{\min}$. This is obtained by setting the differential of Eq. (5) equal to zero and solving for p. To gain insight into the underlying physics, we now make some approximations to the exact analytic expressions. For large n, or more importantly large $n - c$, the term $(1-p)^{n-c} \to e^{-\rho}$ in Eq. (5). Provided that the cost per connection to the hub is not too high, the region containing the minimal shortest path $\bar{\ell}|_{\min}$ will be at a reasonably high ρ. Hence we can neglect the exponential term and differentiate to find the minimum value of $\bar{\ell}$ with $c = knp = k\rho$. It is reasonable to assume that at fixed k, optimal ρ will increase with n like n^x where $0 < x \leq 1$. In particular, one obtains diffusive behavior whereby $x \sim 1/2$. Specifically, $\rho \approx \sqrt{\frac{2n}{k}}$. For a large network (i.e. large n), we have therefore obtained a simple relationship between the number of connections one should introduce in order to create the minimal average shortest path between any two nodes in the network, and the cost per connection to the hub. Now we briefly turn to consider a specific yet physically reasonable example of non-linear costs, in which the costs are taken to depend on the number of *pairs* which are connected via the hub. In particular, we use $c = k(np)^2$. We obtain the analytic relationship $\rho \approx \sqrt[3]{\frac{n}{k}}$ which is the non-linear equivalent of the above result. Obviously, more accurate expressions can be obtained since we know the complete form of the analytic solution – however these are too cumbersome algebraically to be presented here. For linear costs, the lowest value of $\bar{\ell}$ one can achieve is $\bar{\ell}|_{\min} \approx \sqrt{8kn}$. For non-linear costs, the minimal shortest path $\bar{\ell}|_{\min} \approx \sqrt[3]{27kn^2}$. These last results show that the minimal shortest path $\bar{\ell}|_{\min}$ across the network grows like $n^{\frac{1}{2}}$ when we impose linear costs while it grows like $n^{\frac{2}{3}}$ when we put a cost on the number of direct connections between nodes made via the hub (i.e. non-linear costs). Corresponding results for the undirected model can be easily obtained from the equations for the directed model. For example for linear costs $c = knp$ and undirected links, we obtain $\bar{\ell}|_{\min} \approx \sqrt{4kn}$ and $\rho \approx \sqrt{\frac{n}{k}}$ for the minimal shortest path and the optimal connectivity. The present analysis can be extended to multiple hubs, $N \geq 2$. For simplicity, we focus here on the specific example of constant costs and $N = 2$ (i.e. hub P, with nodes connected to it with probability p and hub Q, with nodes connected to it with probability q) where the cost associated with each hub has value

c_p and c_q, with $c_p \geq c_q$. The cost for using *both* hubs is assumed to be infinite. We first consider what happens when $\ell > c_p \geq c_q$. In this case, both hubs may be used and we may therefore write:

$$\begin{aligned} P(\ell < m) = & P_P(\ell < m, \ell > c_p)\big[1 - \sum_{i-c_q=1}^{\ell-c_q-1} P_Q(i < m, i > c_q)\big] \\ & + P_Q(\ell < m, \ell > c_q)\big[1 - \sum_{i-c_p=1}^{\ell-c_p-1} P_P(i < m, i > c_p)\big] \\ & - P_P(\ell < m, \ell > c_p) P_Q(\ell < m, \ell > c_q) \end{aligned} \quad (6)$$

where $P_P(\ell < m, \ell > c_p)$ and $P_Q(\ell < m, \ell > c_q)$ are understood to be $P(\ell < m, \ell > c)$ from the single-hub-with-costs case for probabilities p and q respectively. Substituting Eq. (2) into the first term of Eq. (6) and performing the summation yields:

$$\begin{aligned} & P_P(\ell < m, \ell > c_p)\big[1 - \sum_{i-c_q=1}^{\ell-c_q-1} P_Q(i < m, i > c_q)\big] \\ & \qquad = (g_{0pq} + g_{1pq}\ell + g_{2pq}\ell^2)(a_p a_q)^{\ell-1} \end{aligned} \quad (7)$$

where

$$\begin{aligned} a_p &= 1 - p \\ a_q &= 1 - q \\ g_{0pq} &= (1-p)^{-c_p}(1-q)^{-c_q} p^2 c_p((c_q+1)q - 1) \\ g_{1pq} &= (1-p)^{-c_p}(1-q)^{-c_q} p^2 (1 - (c_p + c_q + 1)q) \\ g_{2pq} &= (1-p)^{-c_p}(1-q)^{-c_q} p^2 q \quad . \end{aligned}$$

An equivalent substitution and summation performed on the second term in Eq. (6) yields the same answer but with labels p and q interchanged. The third term, after substitution and summation, yields:

$$\begin{aligned} & P_P(\ell < m, \ell > c_p) P_Q(\ell < m, \ell > c_q) \\ & \qquad = (h_0 + h_1\ell + h_2\ell^2)(a_p a_q)^{\ell-1} \end{aligned} \quad (8)$$

where

$$\begin{aligned} h_0 &= (1-p)^{-c_p}(1-q)^{-c_q} p^2 q^2 c_p c_q \\ h_1 &= -(1-p)^{-c_p}(1-q)^{-c_q} p^2 q^2 (c_p + c_q) \\ h_2 &= (1-p)^{-c_p}(1-q)^{-c_q} p^2 q^2 \quad . \end{aligned}$$

Substitution of these individual terms into Eq. (6) yields:

$$P(\ell < m) = (g'_0 + g'_1\ell + g'_2\ell^2)(a_p a_q)^{\ell-1} \quad (9)$$

where $g'_i = g_{ipq} + g_{iqp} - h_i$. To calculate the full probability distribution for the case $\ell > c_p \geq c_q$ we now only require $P(\ell = m)$:

$$P(\ell = m) = 1 - \sum_{i=c_q+1}^{c_p} P_Q(i < m) - \sum_{i=c_p+1}^{\ell-1} P(i < m) \quad (10)$$

where $P_Q(i < m)$ is the single-hub-plus-costs distribution for a hub with probability q and $P(i < m)$ is given by Eq. (9). We define the following functions:

$$\begin{aligned} f_x(a,n) &= \sum_{i=1}^{n-1} i^x a^{i-1} \\ \tilde{f}_x(a,n_1,n_2) &= f_x(a,n_1) - f_x(a,n_2) \quad . \end{aligned}$$

We then substitute $P_Q(i < m)$ and $P(i < m)$ into Eq. (10) yielding:

$$\begin{aligned} P(\ell = m, \ell > c_p) = 1 - \frac{q^2}{(1-q)^{c_q}} \big[&\tilde{f}_1(a_q, c_p+1, c_q+1) \\ -c_q \tilde{f}_0(a_q, c_p+1, c_q+1) \big] - \big[&g'_0 \tilde{f}_0(a_p a_q, \ell, c_p+1) \\ +g'_1 \tilde{f}_1(a_q a_p, \ell, c_p+1) + &g'_2 \tilde{f}_2(a_p a_q, \ell, c_p+1) \big] \quad . \end{aligned} \tag{11}$$

We now obtain the final distribution by performing the sum over m:

$$P(\ell, \ell \leq c_q) = \frac{1}{n-1} \tag{12}$$

$$\begin{aligned} P(\ell, c_q < \ell \leq c_p) = \frac{1}{n-1} \big[&1 + (\ell - c_q - 1)q \\ +(n-1-\ell)(\ell - c_q)q^2 \big] &(1-q)^{\ell - c_q - 1} \end{aligned} \tag{13}$$

$$\begin{aligned} P(\ell, c_p < \ell) = \frac{1}{n-1} \Bigg[1 - \frac{q^2}{(1-q)^{c_q}} &\big[\tilde{f}_1(a_q, c_p+1, c_q+1) \\ -c_q \tilde{f}_0(a_q, c_p+1, c_q+1) \big] - &\big[g'_0 \tilde{f}_0(a_p a_q, \ell, c_p+1) \\ +g'_1 \tilde{f}_1(a_q a_p, \ell, c_p+1) + &g'_2 \tilde{f}_2(a_p a_q, \ell, c_p+1) \big] \\ +\big[(n-1-\ell)(g'_0 + g'_1 \ell + &g'_2 \ell^2)(a_p a_q)^{\ell-1} \big] \Bigg] \quad . \end{aligned} \tag{14}$$

These results were used directly to produce the figure and curve shown in Fig. 4C (top) for one platform (universe 1) with one long chain (i.e. one ring) having separate sets of wormholes to a second platform (universe 2) and a third platform (universe 3).

In what follows, we consider the other example in Fig. 4B,C (bottom) of the main paper, of two distinct platforms (universe 1 and universe 2) having separate sets of wormholes to the same third platform (universe 3). Interconnections between the nodes from different platforms are not considered. The only coupling between the two platforms is due to the cost of using the third platform (referred to here as the central hub). Since both the platforms' rings will tend to optimize their average path lengths, it is interesting to see how the optimal number of connections and the minimum path length changes due to the central hub being shared. Let two platform rings A and B have sizes and connecting probability of nodes n, n_B, p and p_B respectively. Considering platform ring A, we start from the expression for a simplified form of the average path length:

$$\bar{l} \approx \frac{p\left(2-2c+2n-(c-1)(c-n)p\right)-3}{p^2(n-1)} + \frac{c(c-1)}{2(n-1)} \tag{15}$$

In this case, the cost depends on the connectivity of both platforms as the congestion is caused by the connections from both the platforms, $c \equiv c(p, p_B)$. For fixed network sizes n and n_B, $\bar{l}$ depends on two variables p and p_B. To find the local maxima or minima, we need to locate the critical points by solving the following simultaneous equations as we require the tangent plane to be horizontal:

$$\frac{\partial}{\partial p}\bar{l}(p, p_B) = \frac{\partial}{\partial p_B}\bar{l}(p, p_B) = 0 \tag{16}$$

where

$$\begin{aligned}
\frac{\partial}{\partial p}\bar{l}(p, p_B) &= 0 \\
&= \frac{\partial}{\partial p}\left[\frac{p\left(2-2c+2n-(c-1)(c-n)p\right)-3}{p^2(n-1)} + \frac{c(c-1)}{2(n-1)}\right] \\
&= -p\left[2-2c+2n-\left(c^2-cn-c+n\right)p\right] \\
&\quad +p^2\left[-2\frac{\partial c}{\partial p}-\left(c^2-cn-c+n\right)-p(2c-n-1)\frac{\partial c}{\partial p}\right]+6+\frac{p^3}{2}(2c-1)\frac{\partial c}{\partial p} \\
&= -p(2-2c+2n)-2p^2\frac{\partial c}{\partial p}-p^3(2c-n-1)\frac{\partial c}{\partial p}+6+\frac{p^3}{2}(2c-1)\frac{\partial c}{\partial p}
\end{aligned} \tag{17}$$

Rewriting in terms of the scaled connectivity, $\rho = np$:

$$\frac{2\rho}{n}(1-c+n)-6 = \frac{\partial c}{\partial p}\frac{\rho^3}{n^2}\left(n+\frac{1}{2}-c-\frac{2n}{\rho}\right) \tag{18}$$

In the limit $n \gg c, \rho \gg 1$,

$$\frac{\partial c}{\partial \rho} = \frac{2n}{\rho^2} \tag{19}$$

Similarly we have,

$$
\begin{aligned}
\frac{\partial}{\partial p_B}\bar{l}\left(p,p_B\right) &= 0 \\
&= \frac{\partial}{\partial p_B}\left[\frac{p\left(2-2c+2n-\left(c-1\right)\left(c-n\right)p\right)-3}{p^2\left(n-1\right)}+\frac{c\left(c-1\right)}{2\left(n-1\right)}\right] \\
&= \frac{1}{2}\left(2c-1\right)\frac{\partial c}{\partial p_B}+\frac{1}{p}\left[-2\frac{\partial c}{\partial p_B}-p\left(2c\frac{\partial c}{\partial p_B}-n\frac{\partial c}{\partial p_B}-\frac{\partial c}{\partial p_B}\right)\right] \\
&= \frac{\partial c}{\partial p_B}\left(\frac{2c-1}{2}-\frac{2}{p}-2c+n+1\right) \\
c &\approx n-\frac{2n}{\rho} \qquad (20)
\end{aligned}
$$

The above analysis only strictly holds true for $\rho \gg 1, n \gg c$, but should be a reasonable first approximation otherwise. The two networks should be of similar sizes for this optimal condition. Since A and B are just dummy labels for the networks, by symmetry we get a similar equation for network B.

$$
\frac{\partial c}{\partial \rho_B} = \frac{2n_B}{\rho_B^2} \qquad (21)
$$

When the hub is used by a single network, the location of the minimum of average path length is obtained from $\frac{\mathrm{d}c}{\mathrm{d}\rho}=\frac{2n}{\rho^2}$.

For a general cost, $c = k\rho^\alpha + q\rho_B^\theta$, the optimum number of connections for the individual networks remains unchanged. However, the optimal average path length $\bar{l}$ does change. We expect it to increase as the additional cost due to the other network causes path lengths $l \le c$ to be much higher and these do not use the hub, thus causing the overall $\bar{l}$ to increase.

Consider the linear case: for optimized networks, we have

$$
\begin{aligned}
c &= k\rho + q\rho_B \\
&= k\sqrt{\frac{2n}{k}}+q\sqrt{\frac{2n_B}{q}} \\
&= \sqrt{2}\left(\sqrt{kn}+\sqrt{qn_B}\right) \qquad (22)
\end{aligned}
$$

Consider the case when $k = q$ and the network are of same size n. Substituting the optimal number of connections and cost when optimized, and considering the dominant terms, we get:

$$
\begin{aligned}
\bar{l} &\approx \frac{2n}{\rho} + c \\
&= \sqrt{2nk} + \sqrt{2nk} + \sqrt{2n_B q} \\
&= \sqrt{2nk} + \sqrt{2nk} + \sqrt{2nk} \\
&= \sqrt{18nk} \qquad (23)
\end{aligned}
$$

Thus the optimal path length has increased to $\bar{l} \approx \sqrt{18nk}$ as compared to $\bar{l} \approx \sqrt{8nk}$ when the hub is used by a single ring and star network. The above result generalizes to multiple hubs, for linear cost functions and N networks with size n:

$$
\begin{aligned}
\bar{l} &\approx \sqrt{2nk} + (N)\sqrt{2nk} \\
&= \sqrt{(N+1)^2\, 2nk}
\end{aligned} \qquad (24)
$$

Strictly speaking, we require $n \gg c$ for our approximations to be accurate, hence the addition of many hubs might result in the cost being too high for the result to be accurate. One important aspect of the result perhaps is that while the optimal number of connections does not change, the optimal path length does due to the hub being used by other platforms. This feature can be used to detect any platforms that may be using a particular hub without wanting to be detected. Here, since the inter-network connections do not exist and the nodes of this 'dark' platform only connect between themselves, it can only be detected through its influence on the cost associated with the hub. Conversely, if it is known that a platform is using a given hub, given its impact on the path length of other platforms using that hub, the nature of its cost function can be estimated. While the optimal probability remains the same, the path length does increase. This is expected as the paths with a higher cost than the separation of nodes, are avoided and this leads to a higher average path length when the costs are higher.

We can similarly consider a series of platforms (rings) with the outer rings using the inner rings and hub as the renormalized hub. Since our result holds only when $n \ll c$, the assumption breaks down for higher rings when the number of nodes are considered equal for all rings. Therefore we consider the case $n_i = n^i$ and a linear cost function. To check if the assumption holds, consider a general x^{th} ring,

$$
\begin{aligned}
c_x &= k\rho_x \bar{l}_{x-1} \\
&= k\rho_x \sqrt{4n_{x-1}k\sqrt{4n_{x-2}k\sqrt{4n_{x-3}k\ldots\ldots\ldots\ldots}}} \\
&= k\rho_x \left(4k\right)^{\frac{1}{2}+\frac{1}{4}+\frac{1}{8}+\frac{1}{16}+\ldots..+\frac{1}{2^{x-1}}} n^{\frac{x-1}{2}} n^{\frac{x-2}{4}} n^{\frac{x-3}{8}} \ldots\ldots\ldots\ldots\ldots n^{\frac{1}{2^{x-1}}} \\
&= k\rho_x \left(4k\right)^{1-\frac{1}{2^{x-1}}} n^{\sum_{j=1}^{x-1} \frac{x-j}{2^j}} \\
&= k\rho_x \left(4k\right)^{1-\frac{1}{2^{x-1}}} n^{\left(x-2+2^{1-x}\right)} \qquad (25)
\end{aligned}
$$

The dominant term is the one in n for any higher x, $c_x \approx \frac{n^x}{n^2}$ and thus $n_x \gg c_x$ hold true for arbitrary higher x. The summation used is evaluated below.

Let $\mathrm{S}_n = \frac{1}{2} + \frac{1}{4} + \frac{1}{8} + \frac{1}{16} + ... + \frac{1}{2^{x-1}} = \sum_{j=1}^{x-1} \frac{1}{2^j} = 1 - \frac{1}{2^{x-1}}$

$$\begin{aligned} \sum_{j=1}^{x-1} \frac{x-j}{2^j} &= \sum_{j=1}^{x-1} \frac{x}{2^j} - \sum_{j=1}^{x-1} \frac{j}{2^j} \\ &= x\mathrm{S}_n - \sum_{j=1}^{x-1} \frac{j}{2^j} \end{aligned} \tag{26}$$

To evaluate the sum, let

$$\begin{aligned} \mathrm{S}_m &= \frac{1}{2^1} + \frac{2}{2^2} + \frac{3}{2^3} + \frac{4}{2^4} + ...\frac{x-1}{2^{x-1}} \\ 2\mathrm{S}_m &= 1 + \frac{2}{2^1} + \frac{3}{2^2} + \frac{4}{2^3} + ...\frac{x-1}{2^{x-2}} \\ 2\mathrm{S}_m - \mathrm{S}_m = \mathrm{S}_m &= 1 + \frac{1}{2^1} + \frac{1}{2^2} + \frac{1}{2^3} + ... + \frac{1}{2^{x-2}} - \frac{x-1}{2^{x-1}} \\ &= 1 + \mathrm{S}_n - \frac{1}{2^{x-1}} - \frac{x-1}{2^{x-1}} \\ &= 1 + \mathrm{S}_n - \frac{x}{2^{x-1}} \end{aligned} \tag{27}$$

Substituting yields,

$$\begin{aligned} \sum_{j=1}^{x-1} \frac{x-j}{2^j} &= x\mathrm{S}_n - \left(1 + \mathrm{S}_n - \frac{x}{2^{x-1}}\right) \\ &= x - 2 + \frac{1}{2^{x-1}} \end{aligned} \tag{28}$$

Thus the optimized path length of the mth ring which uses inner rings and the central hub as a renormalized hub for cost function which depends linearly on the number of connections and the path length of the inner ring is:

$$\bar{l}_m = \sqrt{4kn_m\sqrt{4kn_{m-1}.......}} = (4k)^{1-\frac{1}{2^m}}\, n^{\left(m-1+2^{-m}\right)} \tag{29}$$

The main conclusions are therefore as follows:

(1) Even though the hub is being used by other networks, the number of connections required by a platform network to reach its optimal average path length does not change. However, the value of the optimal path length does change. Thus any deviance in optimal path length from the expected value can help in inferring the presence of another network that might otherwise go undetected.

(2) The optimized path length of the mth ring in a series of rings with $n_m = n^m$ which uses inner rings and the central hub as a renormalized hub for the cost function and which depends linearly on the number of connections and the path length of the inner ring, is given by:

$$\bar{l}_m = (4k)^{1-\frac{1}{2^m}} \, n^{\left(m-1+2^{-m}\right)} \tag{30}$$

C. Theoretical description for manipulating outbreak of support surrounding malicious matter (Figs. 4D,E,F main paper). This material draws heavily from the reference given in the main paper: P.D. Manrique, M. Zheng, Z. Cao, E.M. Restrepo, N.F. Johnson, Generalized gelation theory describes onset of online extremist support, Phys. Rev. Lett. 121, 048301 (2018). We refer to that paper for demonstrations of good agreement between the core mathematics and approximations that we use, and numerical simulations – and hence numerical justifications for the approximations that we use. We start with a set of rate equations for the concentration $c_k(t)$ of small clumps (i.e. microscopically small clusters) of individuals size k ($k = 1, 2, ...$). These small clumps of individuals may be anywhere online, in some informal community setting (e.g. some people discussing another topic and/or on another platform). As yet, these clumps of individuals have not yet aggregated (i.e. 'gelled') into a large observable malicious matter cluster (e.g. a Facebook Page focused on hate). When it does, that large cluster can be called a gel that emerges out of this soup of microscopic clumps – and represents one of the observable Facebook Pages (clusters). We include heterogeneity among the interacting elements (small clumps of size $k = 1$) and consider that this heterogeneity ultimately dictates the evolution of the aggregation process. A hidden variable x that we for simplicity call 'character', is randomly assigned to each element taken from a given distribution $q(x)$. The interaction is described in terms of the similarity or dissimilarity of the interacting elements. We define the similarity S_{ij} between element i and element j as $S_{ij} = 1 - |x_i - x_j|$, so that elements with alike character have a high similarity and otherwise for a pair of elements with unlike character. We consider that the probability of aggregation for any two individuals i and j is given by $\mathcal{C} = S_{ij}$. Our definition also recognizes the opposite mechanism which tends to form clumps of dissimilar individuals, where the aggregation probability between i and j is $\mathcal{C} = 1 - S_{ij}$. The random case is recovered in the limit where the aggregation probability is independent of x, which is $\mathcal{C} = 1$. The heterogeneous aspect of the aggregation process is transferred by means of a mean-field probability for aggregation. For example, for a uniform character distribution $q(x)$, the probability density function (PDF) of the similarity $y = S_{ij}$, for homophily is $f(y) = 2y$ and hence the mean-field aggregation probability F, becomes:

$$F = \int_0^1 y f(y) dy = 2/3. \tag{31}$$

By contrast, for dissimilarity defining $z = 1 - S_{ij}$, the PDF $f(z) = 2(1 - z)$ resulting into a mean-field aggregation probability F, of:

$$F = \int_0^1 z f(z) dz = 1/3. \tag{32}$$

The homogeneous limit (i.e. random limit) occurs when $y = 1$ (or $z = 0$) and the diversity distribution is a Dirac delta which yields $F = 1$. In general, F determines the likelihood for any pair of elements i and j to merge into a new clump at a given timestep t. With this in mind we can rewrite a set of equations for the number of clumps of size k (n_k), for the

heterogeneous system as:

$$\dot{n}_k(t) = -2F\frac{kn_k}{N^2}\sum_{r=1}^{\infty} rn_r + \frac{F}{N^2}\sum_{r=1}^{k} rn_r(k-r)n_{k-r}, \quad k \geq 2 \tag{33}$$

$$\dot{n}_1(t) = -2F\frac{n_1}{N^2}\sum_{r=1}^{\infty} rn_r, \quad k = 1, \tag{34}$$

where N is the subpopulation from which a particular flavor of future Facebook Page (gel) might emerge if gelation occurs. The first term represents the population of clumps of size k that merge with other clumps, while the second term is the population of smaller clumps that merge to form clumps of size k, consisting of the well-known product kernel. By considering $N = \sum_{r=1}^{\infty} kn_k$, the equation can be immediately solved and the expression for the evolution number of individual elements is:

$$n_1(t) = Ne^{-\frac{2F}{N}t}, \tag{35}$$

where we have assumed that initially the system is comprised by individual elements only, ($n_1(0) = N$). This yields:

$$n_2(t) = Fte^{-\frac{4Ft}{N}}. \tag{36}$$

and the general expression for any $k \geq 2$ is found to be:

$$n_k(t) = \frac{1}{k!}\left(\frac{k}{N}\right)^{k-2}(2Ft)^{k-1}\, e^{-\frac{2kF}{N}t}. \tag{37}$$

For finite systems at some point into the dynamics a finite non-negligible fraction of the total population condenses into a single large cluster. This phenomena is known as gelation and divides the dynamics of the system. Each hate Facebook Page is a large cluster which we can call a gel. After the gel is formed, the moments of the size distribution is decomposed into the small clumps (or *solution*) and the largest gel cluster (Facebook page) in the following way:

$$M_j = \sum_{k\geq 1} k^j n_k = \sum_{sol} k^j n_k + (k^j n_k)_{gel}. \tag{38}$$

The importance of this decomposition becomes evident when analyzing the zeroth moment, $M_0 = \sum_{k\geq 1} n_k$, which provides the number of clumps of any size. By looking at its first derivative we find:

$$\begin{aligned} \frac{dM_0}{dt} &= \sum_{k\geq 1}\frac{dn_k}{dt} \\ &= -2F\sum_k \frac{kn_k}{N} + \frac{F}{N^2}\sum_k\sum_{i+j}^{k}(in_i)(jn_j) \\ &= -F, \quad \Rightarrow \quad M_0(t) = N - Ft. \end{aligned} \tag{39}$$

The solution for the zeroth moment becomes negative when $t > N/F$ which is problematic since M_0 gives the total number of clumps present. This problem is solved by using, above

the gel point, $\sum_{k\geq 1} kn_k = N - C$, where C is the size of the gel cluster (hate Facebook page). With this correction the derivative of the zeroth moment becomes:

$$\begin{aligned}
\frac{dM_0}{dt} &= -\frac{2F}{N}(N-C) + \frac{F}{N^2}(N-C)^2 \\
&= \frac{F}{N}(N-C)\left(\frac{N-C-2N}{N}\right) = -\frac{F}{N^2}(N-C)(N+C) \\
&= \frac{F}{N^2}\left(C^2 - N^2\right). \qquad (40)
\end{aligned}$$

This indicates that the number of clumps stops decreasing when the gel reaches the system size N. The appearance of the gel cluster is mathematically manifested as a singularity in the second moment of the size distribution. The evolution of the second moment is given by:

$$\frac{dM_2}{dt} = \sum_{k\geq 1} k^2 \frac{dn_k}{dt}. \qquad (41)$$

and hence:

$$\begin{aligned}
\frac{dM_2}{dt} &= \sum_{k\geq 1}\left(\frac{k}{N}\right)^2 F \sum_{i+j=k} in_i jn_j - \sum_{k\geq 1} k^3 n_k \frac{2F}{N} \\
&= \sum_{\substack{i\geq 1\\ j\geq 1}} (i+j)^2 \frac{F}{N^2} in_i jn_j - \sum_{k\geq 1} k^3 n_k \frac{2F}{N} \\
&= \sum_{\substack{i\geq 1\\ j\geq 1}} \left(i^3 jn_i n_j + 2i^2 j^2 n_i n_j + ij^3 n_i n_j\right)\frac{F}{N^2} - \sum_{k\geq 1} k^3 n_k \frac{2F}{N} \\
&= \sum_{\substack{i\geq 1\\ j\geq 1}} \left(i^2 n_i\right)\left(j^2 n_j\right)\frac{2F}{N^2} + \left(i^3 n_i jn_j + in_i j^3 n_j\right)\frac{F}{N^2} - \sum_{k\geq 1} k^3 n_k \frac{2F}{N} \\
&= \sum_{\substack{i\geq 1\\ j\geq 1}} \left(i^2 n_i\right)\left(j^2 n_j\right)\frac{2F}{N^2} + \sum_{k\geq 1} k^3 n_k \frac{2F}{N} - \sum_{k\geq 1} k^3 n_k \frac{2F}{N} \\
&= \sum_{\substack{i\geq 1\\ j\geq 1}} \left(i^2 n_i\right)\left(j^2 n_j\right)\frac{2F}{N^2} \\
&= M_2^2 \frac{2F}{N}, \qquad (42)
\end{aligned}$$

which gives a closed differential equation for the second moment of the size distribution. The solution for the initial condition where all clumps are of size one ($M_2(0) = N$), is:

$$M_2(t) = \left(\frac{1}{N} - \frac{2Ft}{N^2}\right)^{-1}, \qquad (43)$$

which has a singularity at the time $t_c = N/2F$, showing the point where the gel transition takes place. This critical time depends on the mean-field aggregation probability F and

hence in the formation process. For a uniform character distribution, unlike clusters (dissimilarity) are slower to be formed and hence the transition occurs at a later time than alike cluster formation (homophily). Random clusters are the quickest to form since they have the maximum mean-field aggregation probability per timestep ($F = 1$). The expression for the evolution of the gel size (e.g. hate Facebook Page) is obtained by means of the exponential generating function $\mathcal{E}(y,t) \equiv \sum_{k\geq 1} k n_k e^{yk}$. Hence:

$$\begin{aligned}
\frac{\partial \mathcal{E}}{\partial t} &= \sum_{k\geq 1} k \frac{\partial n_k}{\partial t} e^{yk} \\
&= -\frac{2F}{N}\sum_k k^2 n_k e^{yk} + \frac{F}{N^2}\sum_{i\geq 1}\sum_{j\geq 1}(i+j) i n_i j n_j e^{yk} \\
&= -\frac{2F}{N}\sum_k k^2 n_k e^{yk} + \frac{F}{N^2}\sum_i i^2 n_i e^{yi}\sum_j j n_j e^{yj} + \frac{F}{N^2}\sum_i i n_i e^{yi}\sum_j j^2 n_j e^{yj} \\
&= \frac{2F}{N^2}\mathcal{E}\frac{\partial \mathcal{E}}{\partial y} - \frac{2F}{N}\frac{\partial \mathcal{E}}{\partial y} \\
&= \frac{\partial \mathcal{E}}{\partial y}\frac{2F}{N}\left(\frac{\mathcal{E}}{N} - 1\right). \qquad (44)
\end{aligned}$$

This is known as the inviscid Burgers equation which is the simplest nonlinear hyperbolic equation and can be solved by the method of characteristics. For this type of partial differential equation, the characteristics are straight lines in the y-t plane where $\mathcal{E}$ is constant and have slope $\alpha(1-\mathcal{E}')$, where for simplicity we have defined $\alpha = 2F/N$ and $\mathcal{E}' = \mathcal{E}/N$. The equation of motion for y along the characteristic is therefore:

$$\frac{dy}{dt} = \alpha(1-\mathcal{E}'). \qquad (45)$$

Since $\mathcal{E}$ (and hence $\mathcal{E}'$) is constant, the solution for $y(t)$ along the characteristic is:

$$y(t) = \alpha(1-\mathcal{E}')t + f(\mathcal{E}), \qquad (46)$$

where $f(\mathcal{E})$ depends on the initial conditions which for the generating function we find it to be $\mathcal{E}(y, t=0) = Ne^y$ which yield $y(t=0) = \ln \mathcal{E}'$. The derivation moves forward as follows:

$$\begin{aligned}
y &= \ln \mathcal{E}' + \alpha t(1-\mathcal{E}) \\
e^y &= \mathcal{E}' e^{\alpha t(1-\mathcal{E}')} \\
e^{y-\alpha t} &= \mathcal{E}' e^{-\alpha t \mathcal{E}'}. \qquad (47)
\end{aligned}$$

Now note that the generating function for $y = 0$ yields $\mathcal{E}(0,t) = N - C$ above the gel point and the following expression for the largest cluster is found:

$$\frac{C}{N} = 1 - e^{-\frac{2Ft}{N^2}C}. \qquad (48)$$

The solution can by written by means of the W-Lambert function as:

$$C = N(1 - W(ze^z)/z), \quad z = -2Ft/N. \qquad (49)$$

which is the equation given in the main paper.

D. Example of LDA topic analysis of hate multiverse content before COVID-19 outbreak
WARNING: THIS SECTION CONTAINS OFFENSIVE MATERIAL

Figure S2 shows the typical results that we get from the analysis of the text, as discussed in the main paper.

- Topic: 0
  - Words: 0.072*"white" + 0.028*"jew" + 0.026*"black" + 0.019*"peopl" + 0.013*"jewish" + 0.013*"race" + 0.012*"like" + 0.011*"christian" + 0.009*"german" + 0.009*"muslim"
- Topic: 1
  - Words: 0.027*"right" + 0.023*"trump" + 0.015*"israel" + 0.013*"vote" + 0.010*"leav" + 0.010*"state" + 0.010*"support" + 0.009*"peopl" + 0.009*"fight" + 0.008*"govern"
- Topic: 2
  - Words: 0.114*"fuck" + 0.051*"nigger" + 0.028*"faggot" + 0.024*"retard" + 0.023*"kike" + 0.023*"like" + 0.022*"shit" + 0.020*"look" + 0.009*"stupid" + 0.009*"trump"
- Topic: 3
  - Words: 0.014*"say" + 0.010*"polit" + 0.009*"parti" + 0.008*"school" + 0.007*"trump" + 0.007*"report" + 0.007*"public" + 0.007*"question" + 0.007*"elect" + 0.006*"power"
- Topic: 4
  - Words: 0.017*"countri" + 0.011*"live" + 0.011*"year" + 0.010*"money" + 0.010*"peopl" + 0.010*"work" + 0.010*"like" + 0.007*"go" + 0.007*"america" + 0.007*"china"
- Topic: 5
  - Words: 0.019*"kill" + 0.018*"shoot" + 0.015*"flag" + 0.012*"epstein" + 0.011*"shit" + 0.010*"fuck" + 0.009*"thank" + 0.008*"messag" + 0.008*"check" + 0.007*"pedo"
- Topic: 6
  - Words: 0.018*"peopl" + 0.009*"think" + 0.008*"human" + 0.008*"thing" + 0.007*"world" + 0.007*"like" + 0.006*"need" + 0.006*"know" + 0.005*"differ" + 0.005*"exist"
- Topic: 7
  - Words: 0.032*"like" + 0.030*"women" + 0.013*"want" + 0.013*"girl" + 0.012*"children" + 0.011*"kid" + 0.010*"woman" + 0.009*"sound" + 0.008*"child" + 0.008*"look"
- Topic: 8
  - Words: 0.023*"post" + 0.022*"know" + 0.017*"good" + 0.016*"thread" + 0.015*"time" + 0.014*"like" + 0.013*"go" + 0.013*"thing" + 0.012*"anon" + 0.011*"shit"
- Topic: 9
  - Words: 0.014*"believ" + 0.012*"second" + 0.011*"jesus" + 0.011*"say" + 0.010*"like" + 0.007*"know" + 0.007*"christ" + 0.006*"come" + 0.006*"read" + 0.005*"word"

Top 100 words in dataset

Generated from commit: fe94433

```
('fuck', 12850), ('like', 12672), ('peopl', 10164), ('dont', 8464), ('white', 7276),
('think', 6967), ('know', 6765), ('shit', 5971), ('want', 5608), ('go', 5426),
('time', 5352), ('nigger', 5026), ('look', 4676), ('thing', 4414), ('right', 4357),
('good', 4350), ('say', 4341), ('your', 4263), ('year', 4231), ('need', 3940),
('that', 3793), ('work', 3783), ('come', 3663), ('trump', 3505), ('jew', 3456),
('countri', 3427), ('actual', 3388), ('live', 3367), ('post', 3270), ('women', 3203),
('mean', 3125), ('world', 3065), ('retard', 3057), ('get', 2781), ('happen', 2774),
('kill', 2695), ('black', 2652), ('tell', 2622), ('faggot', 2618), ('liter', 2589),
('base', 2417), ('leav', 2395), ('make', 2388), ('state', 2323), ('doesnt', 2290),
('start', 2280), ('thread', 2280), ('stop', 2272), ('better', 2251), ('american',
2212), ('life', 2200), ('real', 2147), ('theyr', 2136), ('tri', 2127), ('didnt',
2116), ('point', 2090), ('kike', 2077), ('take', 2040), ('believ', 2039), ('talk',
1956), ('isnt', 1934), ('love', 1865), ('anon', 1841), ('money', 1827), ('sure',
1816), ('power', 1762), ('care', 1760), ('read', 1725), ('probabl', 1717), ('nation',
1715), ('give', 1700), ('america', 1686), ('race', 1649), ('reason', 1620), ('hate',
1615), ('feel', 1611), ('yeah', 1588), ('govern', 1581), ('christian', 1577),
('person', 1574), ('human', 1568), ('vote', 1567), ('place', 1542), ('jewish', 1537),
('problem', 1513), ('there', 1509), ('call', 1508), ('pretti', 1501), ('chang', 1488),
('differ', 1486), ('wrong', 1471), ('girl', 1471), ('long', 1468), ('true', 1455),
('understand', 1401), ('israel', 1384), ('stupid', 1379), ('kid', 1377), ('lose',
1372), ('great', 1368)
```

Figure S2: Example output from our machine learning (LDA) analysis discussed in the main paper text.

E. Direct and indirect links between hate clusters in different social networks

Source	Target					
	4Chan	Facebook	Gab	Instagram	Telegram	VKontakte
4Chan	-	0	17	0	214	0
Facebook	0	-	0	0	221	18
Gab	465	3	-	0	169	0
Instagram	0	0	0	-	70	0
Telegram	473	217	320	77	-	42
VKontakte	2	210	2	4	1,742	-

Table 1 Direct links between hate clusters in different social networks. It lists *one-step* (i.e. direct) hyperlinks between social networks connecting hate clusters from the Source (which can be regarded in this sense as a broadcasting) platform and the Target (or in this sense, receiving) platform, as shown visually in Fig. 1 in the main article. The key connective role of Telegram, and to lesser extent VKontakte, is evident in how they are the two platforms that receive and broadcast to all other 5 platforms. Links between hate clusters of the same platform are not shown.

Source		Target					
Origin	*Intermediate*	4Chan	Facebook	Gab	Instagram	Telegram	VKontakte
4Chan	Gab	-	1	0	0	18	0
4Chan	Telegram	-	17	0	5	0	0
Facebook	Telegram	0	-	0	0	0	1
Gab	4Chan	0	0	-	0	663	0
Gab	Telegram	14	13	-	0	0	1
Instagram	Telegram	11	21	0	-	0	0
Telegram	4Chan	0	0	310	0	-	0
Telegram	Gab	5	3	0	0	-	0
Telegram	VKontakte	0	2	1	1	-	0
VKontakte	4Chan	0	0	5	0	51	-
VKontakte	Facebook	0	0	0	0	2	-
VKontakte	Telegram	6	51	3	3	0	-

Table 2 Indirect links between hate clusters in different social networks, analogous to Table 1 above. This table lists *two-step* (i.e. indirect) mediated connections, describing how many paths we found from a cluster in an Origin platform that links into a cluster in an Intermediate platform (1st step) and which could then reach a third cluster in a Target platform (2nd Step).
